# Impact of Cultivation and Origin on the Fruit Microbiome of Apples and Blueberries and Implications for the Exposome

**DOI:** 10.1007/s00248-022-02157-8

**Published:** 2022-12-21

**Authors:** Wisnu Adi Wicaksono, Aisa Buko, Peter Kusstatscher, Tomislav Cernava, Aki Sinkkonen, Olli H. Laitinen, Suvi M. Virtanen, Heikki Hyöty, Gabriele Berg

**Affiliations:** 1 Institute of Environmental Biotechnology, Graz University of Technology, Graz, Austria; 2 Natural Resources Institute Finland Luke, Turku, Finland; 3 Faculty of Medicine and Health Technology, Tampere University, Tampere, Finland; 4 Health and Well-Being Promotion Unit, Finnish Institute for Health and Welfare, Helsinki, Finland; 5 Faculty of Social Sciences, Unit of Health Sciences, Tampere University, Tampere, Finland; 6 Research, Development and Innovation Center, Tampere University Hospital, Tampere, Finland; 7 Center for Child Health Research, Tampere University and Tampere University Hospital, Tampere, Finland; 8 Fimlab Laboratories, Pirkanmaa Hospital District, Tampere, Finland; 9 Leibniz Institute for Agricultural Engineering and Bioeconomy (ATB), Potsdam, Germany; 10 Institute for Biochemistry and Biology, University of Potsdam, Potsdam, Germany

**Keywords:** Fruit microbiome, Apple, Blueberry, Amplicon sequencing, Growing system, Naturally grown, Commercial horticulture

## Abstract

Vegetables and fruits are a crucial part of the planetary health diet, directly affecting human health and the gut microbiome. The objective of our study was to understand the variability of the fruit (apple and blueberry) microbiome in the frame of the exposome concept. The study covered two fruit-bearing woody species, apple and blueberry, two countries of origin (Austria and Finland), and two fruit production methods (naturally grown and horticultural). Microbial abundance, diversity, and community structures were significantly different for apples and blueberries and strongly influenced by the growing system (naturally grown or horticultural) and country of origin (Austria or Finland). Our results indicated that bacterial communities are more responsive towards these factors than fungal communities. We found that fruits grown in the wild and within home gardens generally carry a higher microbial diversity, while commercial horticulture homogenized the microbiome independent of the country of origin. This can be explained by horticultural management, including pesticide use and post-harvest treatments. Specific taxonomic indicators were identified for each group, i.e., for horticultural apples: *Pseudomonas*, *Ralstonia*, and *Stenotrophomonas*. Interestingly, *Ralstonia* was also found to be enriched in horticultural blueberries in comparison to such that were home and wildly grown. Our study showed that the origin of fruits can strongly influence the diversity and composition of their microbiome, which means that we are exposed to different microorganisms by eating fruits from different origins. Thus, the fruit microbiome needs to be considered an important but relatively unexplored external exposomic factor.

**Supplementary Information:**

The online version contains supplementary material available at 10.1007/s00248-022-02157-8.

## Introduction

Vegetables and fruits are a crucial part of the planetary health diet EAT-Lancet Commission [1]. This diet aims to simultaneously provide health to the population and the planet as required by the Food and Agriculture Organization (FAO) and World Health Organization [2]. In addition to vegetables and fruits, this diet is based on the predominant consumption of greens and whole grains, and reduced consumption of meat, fish, eggs, refined cereals, and tubers [3]. Vegetables and fruits contain various ingredients and bioactive plant-derived secondary metabolites which are considered to have beneficial health effects. Moreover, they harbor millions of microorganisms [4], which potentially serve as one of the main direct sources of environmental microbiota. The human gut microbiome is regarded as an internal environmental factor, while the impact of the external environment, including the food microbiota, is less well understood in the exposome concept. The exposome concept was first suggested by Wild [5] to encompass the totality of human environmental exposures from conception onwards, complementing the genome. The concept differentiates three categories of non-genetic exposures: internal, specific external, and general external [6]. Recently, intervention trials to demonstrate the importance of external, nature-based microbe exposures on the human microbiota and immune functions were reported [7, 8].

as well as factors that shape them are still scarce. During the last years, an accumulating amount of evidence has shown that fruits are colonized by distinct microbial communities. Studies have examined the impact of host genetics [9] and environmental influences, e.g., soil and climate [10, 11] on the composition of the fruit microbiota. Moreover, post-harvest treatments, such as washing, waxing, storage, and thermal treatment, were shown to strongly influence the composition of fruit microbiota [12–14]. Recently, the beneficial effects of fruit consumption on the gut microbiota and human health have been increasingly recognized [15, 16]. However, our knowledge of the microbial composition of fruits produced in different growing systems and geographic regions is still very limited.

Despite the importance of the fruit and vegetable associated microbiota, studies on fresh fruits and vegetables. Our objective was to understand the fruit microbiome in the context of the exposome concept. We expected that the fruit microbiome is an important external factor that influences the gut microbiome especially during the early life. First, however, it is important to understand the variability of the fruit microbiome between different growing systems. Therefore, we have selected apples and blueberries, which are among the most commonly consumed raw fruits in the world and, more importantly, are commonly eaten in early childhood. Apples can be grown in home gardens as well as in commercial orchards. Blueberries are grown in commercial farms and can be also found in the wild. These different growing systems make both fruits ideal models to further study the variability of the fruit microbiota that are commonly consumed. The growing systems are characterized by different management practices. While no chemicals, nor fertilization were used in fruits that were grown in the wild and within home gardens, typical horticultural systems are intensively treated. In this study, we attempted to address the following questions: (i) do fruits of natural origin have a different microbial diversity compared to horticulturally grown fruits; (ii) are there differences in the microbial composition between these two groups; (iii) are there differences between fruits from distinct geographical locations; and (iv) which taxa explain the differences between the two groups? Overall, this study provides important insights into the impacts of growing systems on the apple and blueberry microbiota.

## Materials and Methods

### Sampling Procedures and DNA Extraction

Apple (*Malus domestica*) and blueberry samples were collected between July and August 2020 at 29 locations (Austria — 20 locations; Finland — 9 locations, Supplementary Table S1) in Austria and Finland. We chose these countries to test if geographic distance had an effect of fruit microbiota compositions. Here, we have defined naturally grown fruits as those grown in the wild or in private gardens, away from commercial orchards, and have not undergone any post-harvest treatment. A total of 15 and 6 naturally grown apple and blueberry samples, respectively, were collected in Austria while a total of 5 and 4 naturally grown apple and blueberry samples, respectively, were collected in Finland. The naturally grown apple and blueberry samples were collected from ecologically isolated individuals. Ripe fruits were collected using sterile gloves and instruments. We randomly selected at least two apple fruits per sampling tree. For blueberry samples, we collected four composite samples (containing at least 10 berries) from four adjacent bushes/shrubs in one location. All fruits that represented horticultural production, were obtained from local supermarkets in Austria and Finland. We decided to obtain fruits from the local supermarket because it is the point that fruits are purchased and consumed. It should be noted that, in this study, most of the naturally grown blueberries belong to *Vaccinium myrtillus* whereas horticultural blueberries mostly belong to *Vaccinium corymbosum* (Supplementary Table S1). Sampling was carried out by using hand gloves and changing the hand gloves between handling various samples. All samples were put in sterile bags, kept in a cooling box during transportation, and stored at 4 °C before processing. Upon arrival in the laboratory, all samples were processed under sterile conditions. A total of 108 apple samples and 100 blueberry samples were analyzed. Details related to the samples and the associated metadata are presented in Supplementary Table S1.

To extract microorganisms from the fruits, approx. 10 g of each fruit sample was homogenized in a BagMixer laboratory blender (Interscience, Saint-Nom-la-Bretèche, France) with 10 ml sterile NaCl (0.85%) solution for 3 min. A total of 2 ml of homogenized suspensions was then centrifuged for 20 min at 16,000 g and pellets were used for DNA extraction. DNA extraction was carried out using the FastDNA SPIN Kit for soil and the FastPrep Instrument (MP Biomedicals, Santa Ana, CA, USA) according to the manufacturer’s protocol. DNA quality and yield were determined using the Nanodrop 2000 UV–Vis spectrophotometer (Thermo Fisher Scientific Inc., Waltham, MA, USA) and then stored at − 20 °C for further PCR reactions.

### Bacterial and Fungal Quantification Using Quantitative Real-Time PCR (qPCR)

By implementing a qPCR-based analysis, we first calculated microbial abundance in the fruit samples (copies maker genes/gram). The qPCR analysis was based on SYBR Green fluorescence using KAPA SYBR FAST qPCR Kit (Kapa Biosystems, Woburn, USA) using the primer pair 515f–806r [17] and ITS1f-ITS2r [18] for bacterial and fungal quantification, respectively. The qPCR reactions and standard preparations were conducted as described previously [19]. Fluorescence quantification was performed using the Rotor-Gene 6000 real-time rotary analyzer (Corbett Research, Sydney, Australia) with initial denaturation at 95 °C for 10 min, followed by 40 cycles of denaturation at 95 °C for 30 s, annealing at 54 °C for 30 s, and extension at 72 °C for 30 s and a final melting curve. The calculated PCR efficiencies were in a range of 94–98% (*R*^2^ = 0.955–0.965) for 515f–806r primers and 80–88% (*R*^2^ = 0.993–0.995) for ITS1f-ITS2r primers.

### 16S rRNA Gene Fragment and Internal Transcript Spacer (ITS) PCR Amplification and Illumina Sequencing

A one-step PCR approach using primers 515F/806R [17] and ITS1f-ITS2r [18] was employed for targeted amplification of the bacterial 16S rRNA V4 region and fungal ITS1 region. The primers contained Illumina indexes (barcode sequences) for multiplexing. Peptide nucleic acid (PNA) clamps were included in the PCR mix to block amplification of the plant’s plastid and mitochondrial DNA. To verify successful amplification, PCR products were visualized on 1% agarose gels and subsequently combined and purified using the Wizard® SV Gel and PCR Clean-Up kit (Promega). All barcoded amplicons were pooled in equimolar concentrations. The pooled samples were sequenced by the commercial sequencing provider Eurofins (Ebersberg, Germany) using the Illumina MiSeq platform (2 × 300 bp paired end reads). Amplicon sequences were deposited at the European Nucleotide Archive (ENA) under the project number PRJEB51939.

### Bioinformatic and Statistical Analysis

Cutadapt was used to remove low quality reads, primer sequences and demultiplex the reads according to the assigned barcode [20]. The DADA2 algorithm [21] was executed in QIIME2 [22] to quality filter, denoise, and remove chimeric sequences. The resulting representative sequences, known as amplicon sequences variants (ASVs), were further classified using the vsearch algorithm against the SILVA v132 and UNITE v7.1 database [23–25].

Statistical analysis and graph rendering were conducted in R studio version 2021.09.0 [26] unless stated otherwise. Prior to statistical analysis, a normality test was performed using the Shapiro test. The data were not normally distributed; therefore, the non-parametric Kruskal–Wallis test was carried out to determine significant differences (*P* < 0.05) of bacterial gene copy numbers per gram of fruits countries and growing systems (commercial versus wild/home-grown). Groups were compared using Dunn’s test of multiple comparisons and the *P* values were adjusted using Benjamini–Hochberg procedure. ASV tables and taxonomic classifications that were generated with the DADA2 algorithm were used as an input for bacterial community analysis. The bacterial community analysis was performed using the software packages Phyloseq and MicrobiomeAnalyst [27, 28]. After removing non-bacterial reads, i.e., mitochondrial and chloroplast sequences, the overall bacterial community, assessed by 16S rRNA gene fragment amplicon sequencing, contained 1,045,695 sequences from the apple dataset and 1,640,743 from the blueberry dataset. These sequences were assigned to 1659 bacterial ASVs. The overall fungal community, assessed by ITS gene fragment amplicon sequencing, contained 911,875 sequences from the apple dataset and 1,896,835 from the blueberry dataset. These sequences were assigned to 1778 fungal ASVs.

The amplicon sequencing datasets were normalized by randomly selecting subsets of sequences (1000 reads for bacterial datasets and 400 reads for fungal datasets). A total of one (16S rRNA dataset) and five (ITS dataset) samples had to be removed due to low read numbers. Respective rarefraction curves indicate that the sampling size was sufficient to capture overall bacterial and fungal diversity (Supplementary Fig. S1). Taxonomical compositions at family level were summarized using bar plots. Based on normalized datasets, differences in microbial alpha diversity (Shannon diversity index – H’) were assessed using the Kruskal–Wallis test. For beta diversity analysis, Bray–Curtis dissimilarity matrices were generated from normalized datasets. We used the *betadisper* function in the vegan package to calculate the distance of each sample to the respective group centroid from the Bray–Curtis distance matrix. The matrix distances were further subjected to analysis of similarities (ANOSIM) to test for significant effects of experimental factors on microbial community structures. Finally, microbial biomarkers at bacterial genus level for horticultural and naturally grown fruits were identified using a linear discriminant analysis effect size (LefSe) analysis [29].

## Results

### Microbial Abundance, Diversity, and Community Structures Were Influenced by Growing Systems and the Country of Origin

**Fig. 1:**
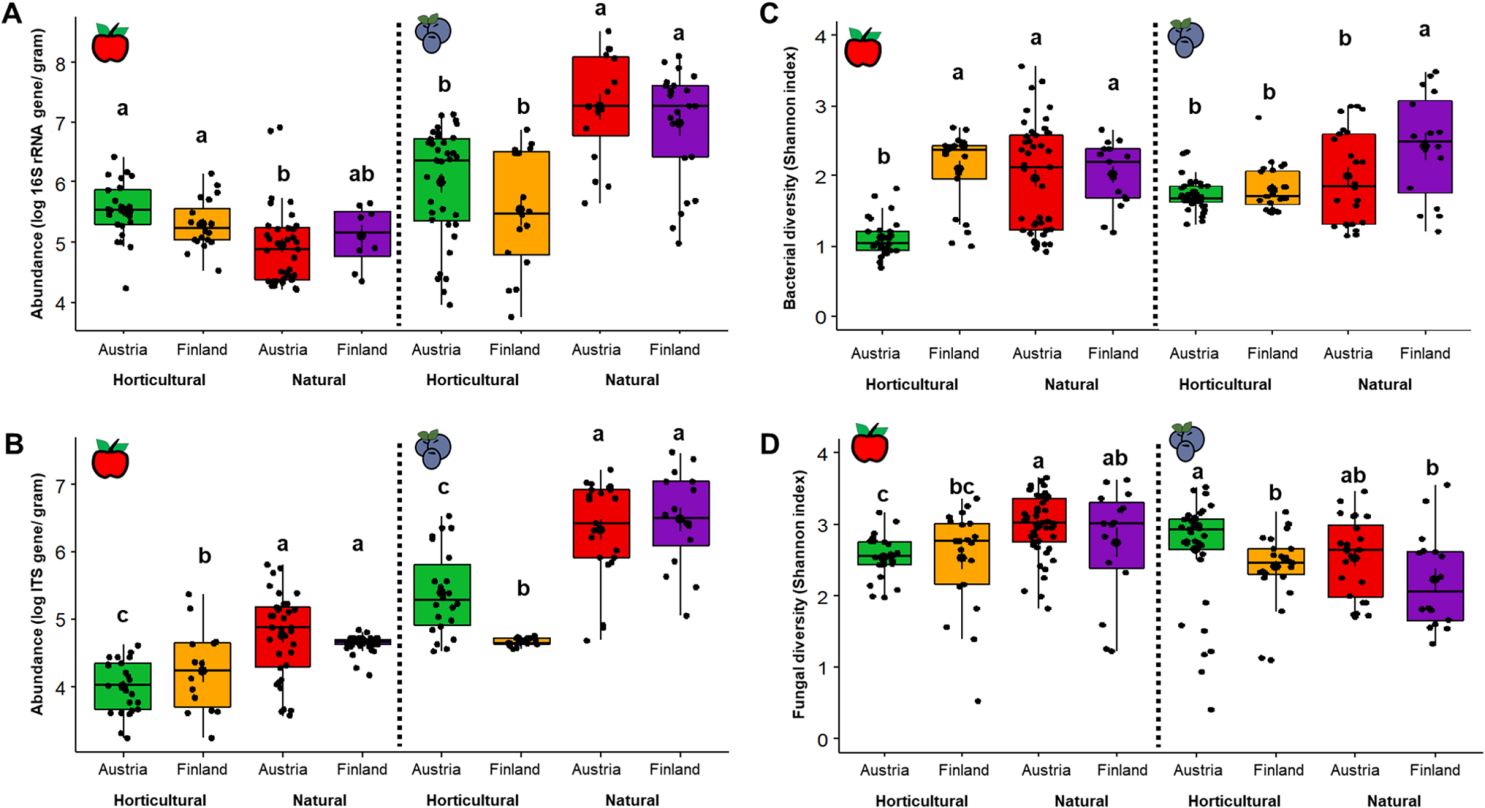
Comparison of microbial abundance and diversity between naturally grown and horticultural fruits. The box plots visualize the variability of microbial abundance (**A** and **B**) and diversity (**C** and **D**). Bacterial (**A**) and fungal (**B**) abundances were quantified using a qPCR-based approach and transformed into log values. In order to compare bacterial (**C**) and fungal (**D**) alpha diversity, the Shannon index was calculated for each sample type. The different letters above each bar indicate statistical significance at *P*_adj_ < 0.05 based on Dunn’s test of multiple comparisons

We observed a significantly higher bacterial and fungal abundance in blueberry samples (copy number of genes/grams: bacterial 2.4 × 10^6^ and fungal 3.7 × 10^5^) in comparison to apple samples (bacteria: 1.6 × 10^5^ and fungi: 2.7 × 10^4^, Kruskal–Wallis test, *P* < 0.05; Fig. 1A and B). A higher bacterial abundance was generally observed in horticultural apples in comparison to naturally/home grown apples whereas the opposite pattern was observed in blueberry samples (Kruskal–Wallis test, *P*_adj_ < 0.05; Fig. 1A). Interestingly, a higher fungal abundance was consistently detected in naturally/home grown apples and blueberries in comparison to the horticultural fruits (Kruskal–Wallis test, *P* < 0.05; Fig. 1B).

By using 16S rRNA and ITS genes fragment amplicon sequencing, we further compared bacterial and fungal diversity between wild/home grown and horticultural fruits. Wild/home grown apples that were obtained in Austria had a higher bacterial and fungal diversity (bacteria: *H*’ = 2.1; fungi: *H*’ = 3.0, Kruskal–Wallis test, *P*_adj_ < 0.05) in comparison to horticultural apples that were obtained from the same country (bacteria: *H*’ = 1.3; fungi: *H*’ = 2.5, Fig. 1C). These differences were not observed from apple samples that were obtained in Finland. When comparing blueberry samples (Fig. 1C and D), homegrown/wild blueberries that were obtained in Finland had a higher (Kruskal–Wallis test, *P*_adj_ < 0.05) bacterial diversity (*H*’ = 1.9) in comparison to horticultural ones obtained in Austria (*H*’ = 1.2) or Finland (*H*’ = 1.3).

Based on ANOSIM, the two tested factors, i.e., growing system and country of origin, had substantial effects on the microbial community structures associated with the fruits (*P* < 0.05; Table 1). Differences between horticultural and naturally grown fruits were observed when the comparison was performed from samples that were obtained from the same country. For examples, horticultural apples and blueberries that were obtained from Austria had different bacterial and fungal community structures in comparison to naturally grown apples and blueberries from Austria (*P*_adj_ < 0.05; Supplementary Table S2). Different fungal community structures were also observed between horticultural and naturally grown apples (*P*_adj_ = 0.010) and blueberries (*P*_adj_ = 0.002) that were obtained in Finland (Supplementary Table S2). Moreover, the country of origin also had an effect on bacterial community composition. For instance, horticultural apples and blueberries obtained in Austria had different bacterial and fungal community composition in comparison to samples that were obtained in Finland (*P*_adj_ < 0.05).

**Table 1:** ANOSIM results on the effects of the growing system and country of origin on bacterial community composition

		Factor	R value	*P* value
Bacteria	Apple	Growing system	0.107	0.001
		Country	0.092	0.006
	Blueberry	Growing system	0.178	0.001
		Country	0.147	0.002
Fungi	Apple	Growing system	0.048	0.024
		Country	0.140	0.003
	Blueberry	Growing system	0.323	0.001
		Country	0.141	0.001

### Horticultural Fruits Harbor a More Homogenous Bacterial and Fungal Community Structure Compared to Naturally Grown Fruits

**Fig. 2:**
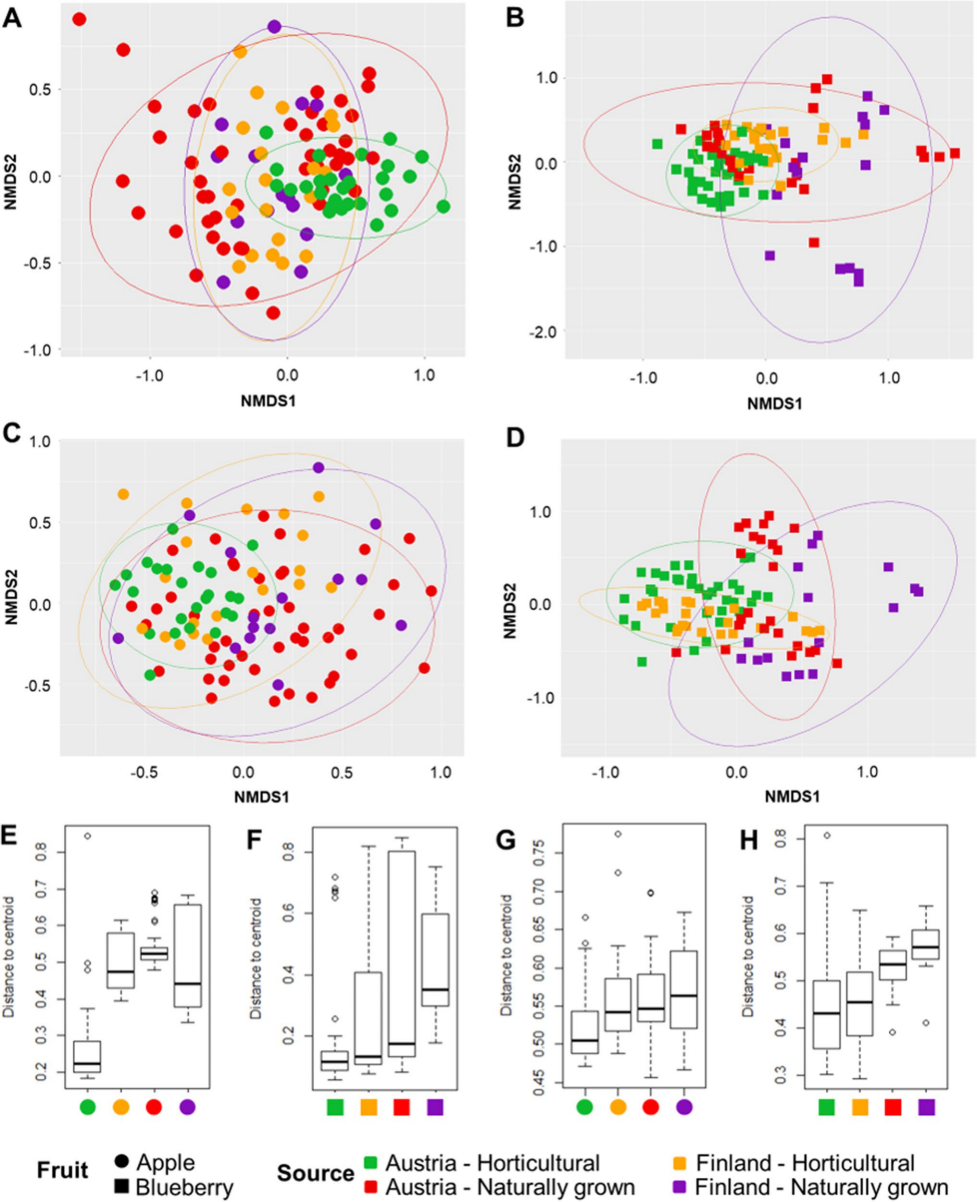
Community clustering and variability in microbial community compositions of naturally grown and horticultural fruits. The nonmetric multidimensional scaling (NMDS) plots show clustering of bacterial (**A** and **B**) and fungal (**C** and **D**) communities from apples and blueberries. The box plots visualize the variability of bacterial (**E** and **F**) and fungal (**G** and **H**) community compositions. Each boxplot shows the distance of the respective sample type to the respective group centroid

NMDS plots supported the obtained ANOSIM results as indicated by a clear clustering of naturally grown and horticultural fruits (Fig. 2). Horticultural apple samples showed a tendency to cluster together whereas naturally grown apple samples were more scattered for both bacterial and fungal datasets (Fig. 2A and C, respectively). This pattern was also more apparent when the apple samples originating from Austria and Finland were analyzed separately (Supplementary Fig. S2A, B and Supplementary Fig. S3A, B). However, the different clustering between horticultural and naturally grown apple were less apparent from the samples that were obtained from Finland. Bacterial and fungal communities from blueberry datasets showed a similar clustering where all the horticultural blueberry samples that were obtained from Austria and Finland clustered together (Fig. 2B and D, Supplementary Fig. S2C and D, and Supplementary Fig. S3C and D).

A complementary analysis using *betadisper* indicated that variability in bacterial and fungal community structures, or the distance of each sample point to the respective group centroid, was higher in naturally grown fruits in comparison to horticultural fruits (Fig. 2E–H). For instance, variability in bacterial communities of horticultural apples (Fig. 2E) that were obtained in Austria was lower (0.276) in comparison to naturally grown apples (0.545). A similar pattern was also observed in the blueberry datasets (Fig. 2F), where a smaller average distance to the centroid was observed in horticultural blueberries (Austria: 0.192 and Finland: 0.285, respectively) compared to naturally grown blueberries (Austria: 0.359 and Finland: 0.419). Similar results were also obtained for the fungal community of blueberries where the variability in fungal communities of naturally grown blueberries was higher in comparison to horticultural blueberries. However, the variability in fungal communities between horticultural and naturally grown apples that were obtained in Austria and Finland was relatively similar (Fig. 2G and H).

### Microbial Community Analysis Revealed Bacterial Indicators Associated with Horticultural and Naturally Grown Fruits

**Fig. 3:**
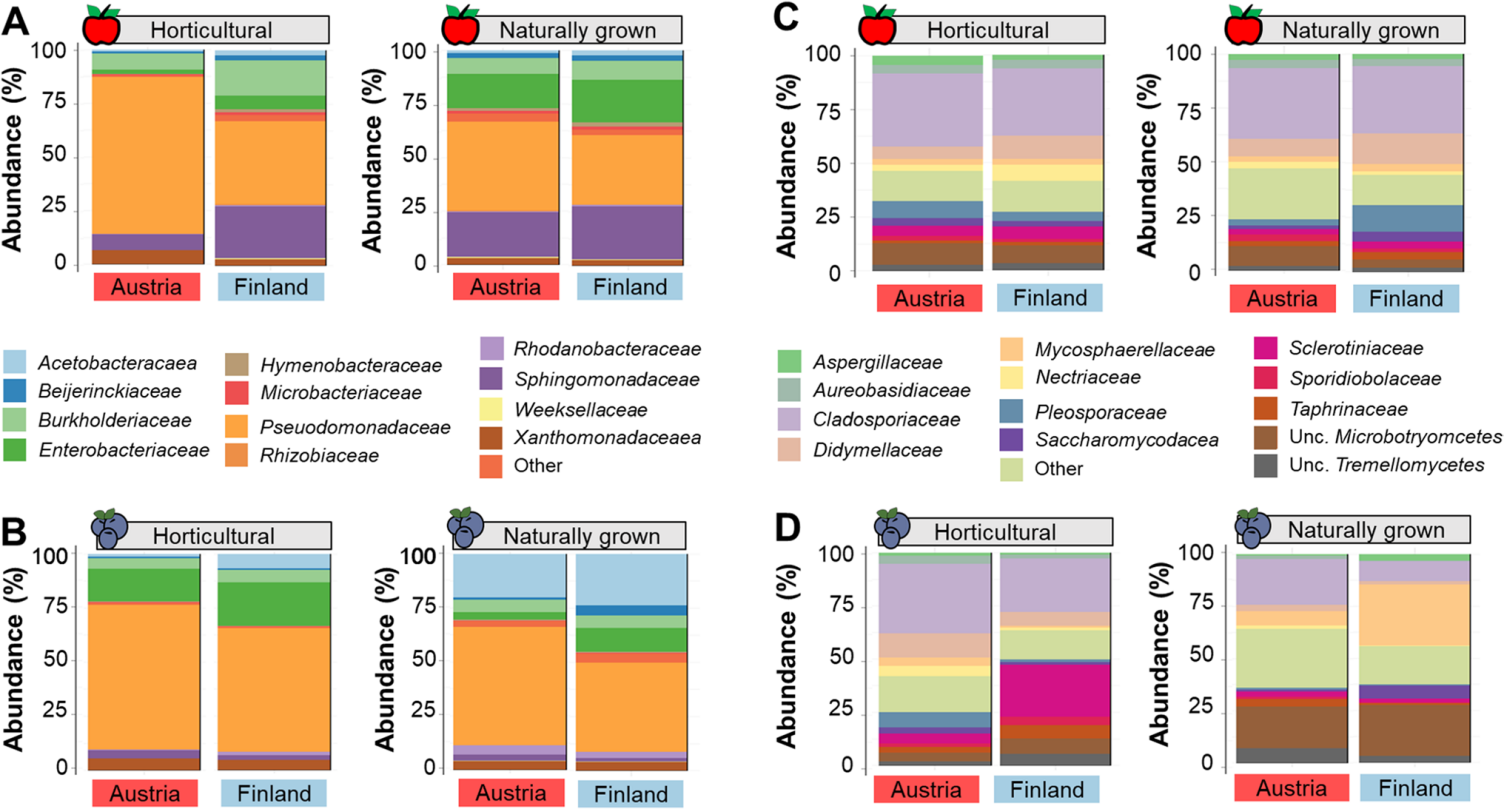
Microbial community composition for naturally grown and horticultural fruits. Bar plots show bacterial (**A** and **B**) and fungal (**C** and **D**) composition at family level for different apple and blueberry samples

To identify bacterial taxa that are specifically enriched in either horticultural or naturally grown fruits, we first visualized the overall bacterial and fungal composition. Apple and blueberry bacterial communities were dominated by *Gammaproteobacteria*, i.e., *Pseudomonadaceae*, *Burkholderiaceae*, and *Enterobacteriaceae* as well as *Alphaproteobacteria*, i.e., *Sphingomonadaceae* (Fig. 3). These taxa contributed 81.5% of the total sequencing reads. A distinct bacterial composition between naturally grown and horticultural apples was indicated by differences in relative abundance of *Pseudomonadaceae*, *Burkholderiaceae*, and *Enterobacteriaceae* (Fig. 3A). Horticultural apples that were obtained from local supermarkets in Austria and Finland harbored a high proportion of *Pseudomonadaceae* and *Burkholderiaceae* that contributed to a total of 80.2 and 55.7% of total reads, respectively with a low abundance of *Enterobacteriaceae* (0.7 and 5.8%, respectively, Fig. 3A). In contrast, the relative abundance of *Enterobacteriaceae* was higher in naturally grown apples that were obtained from both countries (Austrian apples: 15.6% and Finnish apples: 19.6% of total reads). Differences in bacterial community composition between naturally grown and horticultural fruits was also observed for blueberry samples (Fig. 3B). A higher abundance of *Acetobacteraceae* was observed in naturally grown blueberries that were obtained from both countries (Austria: 19.9% and Finland: 23.1%) in comparison to horticultural blueberries (Austria: 1.3% and Finland: 7.3%). In contrast, a higher abundance of *Enterobacteriaceae* was observed in horticultural blueberries (Austria: 15.1% and Finland: 19.8%) in comparison to those naturally grown (Austria: 3.5% and Finland: 11.5%).

Three fungal families, namely *Cladosporiaceae*, *Didymellaceae*, and *Pleosporaceae* were highly abundant in apple samples (49.5%), whereas *Cladosporiaceae*, *Sclerotiniaceae*, and *Mycosphaerellaceae* were highly abundant in blueberry samples (40.9%). The differences in fungal community compositions between horticultural and naturally grown apples were not apparent. A distinct composition was observed for the two countries. For instance, apple samples that were obtained in Finland had a higher abundance of *Didymellaceae* (naturally grown: 14.6% and horticultural: 10.9%) in comparison to apple samples that were obtained in Austria (naturally grown: 8.5% and horticultural: 5.4%, Fig. 3C). In comparison to the apple samples, fungal compositions in blueberries varied more between different groups (Fig. 3D). In horticultural blueberries obtained in Austria and Finland, the relative abundance of *Didymellaceae* (10.6% and 6.3%, respectively) was higher in comparison to naturally grown blueberries that were obtained in the respective countries (*Didymellaceae* 3.0% and 1.6%). In contrast, unclassified members of the fungal class *Microbotryomycetes* were more abundant in naturally grown blueberries (Austria: 18.9% and Finland: 23.4%) in comparison to horticultural blueberries (Austria: 4.4% and Finland: 6.9%).

**Fig. 4:**
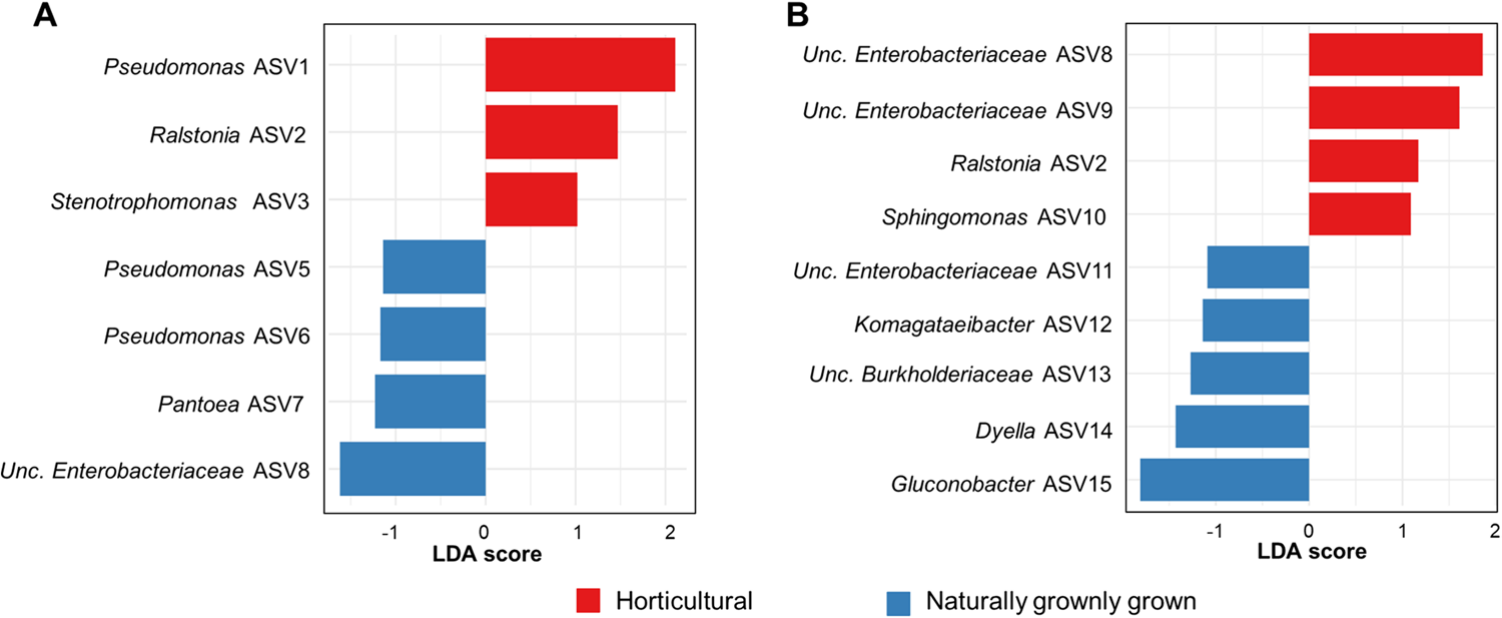
Identification of significantly enriched bacterial taxa in apples and blueberries. Linear discriminant analysis effect size (LEfSe) analysis was implemented to identify microbial biomarkers for naturally grown and horticultural fruits at ASV level. **A** Apple; **B** blueberry

Because differences at higher taxonomic levels may not provide sufficient information to infer ecological relevance, taxa that are indicators for naturally grown or horticultural fruits were identified on ASV level using LEfSe analysis. Three bacterial ASVs, i.e., *Pseudomonas* ASV1, *Ralstonia* ASV2, and *Stenotrophomonas* ASV3 (LDA score > 1, *P* < 0.05) that are closely related to *Pseudomonas mandelii*, *Ralstonia insidiosa*, and *Stenotrophomonas maltophilia*, respectively, were found to be enriched in horticultural apples (Fig. 4A, Supplementary Table S3)*.* Interestingly, *Ralstonia* ASV2 was also found to be enriched in horticultural blueberries in comparison to naturally grown blueberries (Fig. 4A). Three other bacterial ASVs that were further identified as *Tatumella ptyseos* (ASV ID: *Unc. Enterobacteriaceae* ASV8), *Serratia* sp. (ASV ID: *Unc. Enterobacteriaceae* ASV8), and *Sphingomonas aerolata* (ASV ID: *Sphingomonas* ASV10) were also enriched in horticultural blueberries. In contrast, three ASVs that are closely related to *Pseudomonas viridiflava*, *Pseudomonas gingeri*, and *Pantoea agglomerans*, respectively, were enriched in naturally grown apples (Supplementary Table S3). With respect to the blueberry dataset, bacterial ASVs that were enriched in naturally grown berries were *Gluconobacter cerinus* (*Gluconobacter* ASV15), *Dyella japonica* (*Dyella* ASV14), *Robbsia* sp. (Unc. *Burkholderiaceae* ASV13), *Komagataeibacter intermedius* (*Komagataeibacter* ASV12), and *Enterobacter hormaechei* (Unc. *Enterobacteriaceae* ASV11).

Only a low number of fungal ASVs (1 ASV from the apple dataset and 4 ASVs from the blueberry dataset; Supplementary Table S3) were identified as indicator taxa for naturally grown and horticultural fruits. These taxa were only enriched in horticultural fruits in comparison to those grown naturally. One fungal ASV was enriched in both horticultural fruits in comparison to naturally grown fruits and further identified as *Cladosporium cladosporioides* (Supplementary Table S4). Additionally, three fungal ASVs, i.e., Unc. *Sclerotiniaceae* ASV2, *Cladosporium* ASV3, and Unc. *Didymellaceae* ASV4 that were further identified as *Botrytis cinerea*, *Cladosporium allicinum*, and *Epicoccum layuense* were also enriched in horticultural blueberries.

## Discussion

In the present study, we characterized microbial communities of naturally grown and horticultural apples and blueberries in order to identify the impact of the growing system on fruit microbiomes as well as specific signatures for each of the two origins. After subjecting a representative set of samples to various comparative analyses, clear differences in their microbial community compositions were identified suggesting a strong influence of the growing system. We were able to provide insights into the impact of growing systems on the native microbiota consumed with fruits and their potential implications for the exposome.

In detail, we showed that the growing system as well as the country of origin have an impact on microbial abundance and diversity. Previously, it was reported that the type of commercial farming (organic or conventional) affects microbial diversity [4, 30]. However, detailed insights into specific impacts of growing systems remained unresolved so far. Here, we observed that fruits from horticultural production generally carried a lower microbial diversity in comparison to naturally grown fruits. It is known that intensively managed agricultural soils generally have a lower soil biodiversity, while natural ecosystems harbor more complex microbial communities [31, 32]. As soil is widely recognized as a source for microbial communities in the phyllosphere, including fruits [33, 34], it can be assumed that intensive farming practices would not only have an impact on soil microbial diversity but also fruit microbial diversity grown under these growing systems. Detailed insights into impacts of the country of origin and even local environments on microbial communities in apples and other fruits, e.g., grapevine, were provided in foregoing studies [35–37]. The present study confirmed that previously observed patterns also apply for bacterial communities in blueberries which have not been investigated so far. However, variation in the fungal community was explained to a greater extent by these factors. This observation indicates that fungal communities are more responsive towards the growing system and country of origin in comparison to bacterial communities in case of blueberry.

In general, microbial communities from horticultural fruits were observed to be more homogenous in the present study. Commercial farming practice and plant breeding have led to highly productive and consistent quality of fruits [38–40] which is required to meet the consumer demand. Interestingly, these factors also result in profound changes of the microbiome in agricultural crops (reviewed in [41]. Recently, a study by Lupatini and colleague [42] indicated that organic farming systems harbor a more heterogenous soil microbiome than conventional farming systems. As soil is known to be a major source of microbes for aboveground habitats, we speculate that more homogenous microbial communities from fruits produced under commercial management practices could be a result of the more homogenous soil microbiome under this management in comparison to natural soil microbiomes. We hypothesize that this finding is also relevant for human health. Exposure to highly diverse environmental microbiota can shape commensal microbiota and consequently promote immune modulation [8, 43]. In terms of the gut microbiota, microorganisms associated with fresh produce, including fruits, constitute an important exposome. The consumption of fresh fruits, which harbor trillions of microorganisms, is likely influencing the gut microbiota composition. Hence, consuming naturally grown fruits likely exposes our gut to a more diverse environmental microbiota. Recently, Wankhade et al. [44] demonstrated a changed gut microbiome composition following blueberry consumption in mice. They speculated that it is caused by active compounds, e.g., antioxidants in the fruit; however, we suggest that it could also be partially caused by the fruit microbiota. As a growing body of literature suggests that gut microbial diversity is linked to human health [45], it is critical to understand the impact of farming systems on the microbial diversity and composition of the fruits we consume.

In a detailed data assessment, we identified taxonomic indicators for naturally grown and horticultural fruits which include known beneficial plant associated bacteria but also potential human pathogens. For example, *Stenotrophomonas maltophilia*, which is recognized as an emerging global opportunistic pathogen [46], was enriched in horticultural apples. This bacterium was also recently isolated from diseased fruits [47, 48]. Interestingly, *Pantoea agglomerans*, a biocontrol agent against fire blight caused by *Erwinia amylovora* [49], but also a potential opportunistic human pathogen [50], was enriched in naturally grown apples. Presence of potential opportunistic pathogens in fresh produce was reported previously [51–53]. Our results reinforce the notion that fresh produce is a reservoir of opportunistic human pathogens. We also found that an ASV closely related to *Pseudomonas viridiflava*, the causal agent of bacterial shoot blight in apples [54], was enriched in naturally grown apples. This taxon can occur as an endophyte, epiphyte, and saprophyte in both agricultural and natural environments [55]. A similar pattern was also observed in commercial blueberries where putative pathogens and beneficial taxa were present. For instance, *Botrytis cinerea*, a causal agent of gray mold [56, 57], and *Epicoccum layuense*, a potential biocontrol agent against fungal diseases [58], were enriched in commercial blueberries. Due to the fact that we only analyzed healthy fruits, we assume that a balanced proportion of beneficial and pathogenic microorganisms provides resilience towards disease outbreaks as previously shown in seed microbiomes of native plants [59].

It should also be highlighted that although a clear distinction of microbial community structures between commercial and naturally grown fruits was observed, there was a fraction of microbial variation that was explained by the tested factors. Our study does not include data on post-harvest handling, i.e., precooling, packaging, and the storing period that can induce microbial community changes [60, 61]. Interestingly, high abundances of ASVs closely related to *Ralstonia insidiosa* and *Sphingomonas aerolata* that were observed in horticultural fruits might be due to post-harvest handling. *Ralstonia Insidiosa* is a strong biofilm producer and was frequently isolated in various fresh produce [62, 63] whereas *S*. *aerolata* is able to grow at low temperatures [64]. Both traits provide an effective survival strategy during cold storage. Moreover, *P. mandelii* was previously reported to have the ability to grow at low temperature (10–12 °C) due to the cold-adapted physiology of this species [65, 66]. We, therefore, speculate that post-harvest handling could shape and proliferate taxa that are more adaptable towards low temperature and other storage conditions. Moreover, we assume that the observed differences in microbial communities are also additionally affected by other factors including the plant genotype (cultivar and species) and pedoclimatic conditions at the production site [67–69]. To answer this question, targeted studies would be required.

## Conclusion

In conclusion, growing systems were shown to substantially affect the variability of the fruit microbiome. Horticultural production results in a more homogenous fruit microbiome in comparison to naturally grown fruits (wild or home gardens). Moreover, specific changes in the composition of the microbiomes were observed that could have implications for human health. The microbiota associated with fruits and other fresh produce is considered a potential source and a key exposome for the gut microbiota. Hence, consuming naturally grown fruits could potentially expose our gut to diverse microbiota. Moreover, for future research, it is also important to consider the impact of management practices on the indigenous fruit microbiota, an element that is mostly overlooked.

## Supplementary Information

Below is the link to the electronic supplementary material.

Supplementary file1 (DOCX 1630 KB)

## Data Availability

Raw sequencing data for each sample used in this study was deposited at the European Nucleotide Archive (ENA) in the FASTQ format and is available under the Bioproject accession number PRJEB51939.

## Abbreviations

ANOSIM: Analysis of similarities
DNA: Deoxyribonucleic acid
PCR: Polymerase chain reaction
ASV: Amplicon sequences variant
NMDS: Non-metric multidimensional scaling (NMDS)

## References

[1] Hemler EC, Hu FB (2019). Plant-based diets for personal, population, and planetary health. Adv Nutr.

[2] FAO, WHO (2019) Sustainable healthy diets – guiding principles. World Health Organization. https://apps.who.int/iris/handle/10665/329409

[3] Willett W, Rockström J, Loken B, Springmann M, Lang T, Vermeulen S (2019). Food in the Anthropocene: the EAT–Lancet Commission on healthy diets from sustainable food systems. The Lancet.

[4] Wassermann B, Müller H, Berg G (2019). An apple a day: which bacteria do we eat with organic and conventional apples?. Front Microbiol.

[5] Wild CP (2005). Complementing the genome with an “exposome”: the outstanding challenge of environmental exposure measurement in molecular epidemiology. Cancer Epidemiol Prev Biomark.

[6] Wild CP (2012). The exposome: from concept to utility. Int J Epidemiol.

[7] Hui N, Grönroos M, Roslund MI, Parajuli A, Vari HK, Soininen L (2019). Diverse environmental microbiota as a tool to augment biodiversity in urban landscaping materials. Front Microbiol.

[8] Roslund MI, Puhakka R, Grönroos M, Nurminen N, Oikarinen S, Gazali AM et al (2020) Biodiversity intervention enhances immune regulation and health-associated commensal microbiota among daycare children. Sci Adv 6:eaba2578 10.1126/sciadv.aba2578 PMC7556828 33055153

[9] Abdelfattah A, Tack AJ, Wasserman B, Liu J, Berg G, Norelli J (2021). Evidence for host–microbiome co-evolution in apple. New Phytol.

[10] Allard SM, Ottesen AR, Micallef SA (2020). Rain induces temporary shifts in epiphytic bacterial communities of cucumber and tomato fruit. Sci Rep.

[11] Copeland JK, Yuan L, Layeghifard M, Wang PW, Guttman DS (2015). Seasonal community succession of the phyllosphere microbiome. Mol Plant Microb Interact.

[12] Abdelfattah A, Whitehead SR, Macarisin D, Liu J, Burchard E, Freilich S (2020). Effect of washing, waxing and low-temperature storage on the postharvest microbiome of apple. Microorganisms.

[13] Wassermann B, Kusstatscher P, Berg G (2019). Microbiome response to hot water treatment and potential synergy with biological control on stored apples. Front Microbiol.

[14] Wicaksono WA, Buko A, Kusstatscher P, Sinkkonen A, Laitinen OH, Virtanen SM et al (2022) Modulation of the food microbiome by apple fruit processing. Food Microbiol 108:104103 10.1016/j.fm.2022.104103 36088117

[15] Tomova A, Bukovsky I, Rembert E, Yonas W, Alwarith J, Barnard ND (2019). The effects of vegetarian and vegan diets on gut microbiota. Front Nutr.

[16] Trošt K, Ulaszewska MM, Stanstrup J, Albanese D, De Filippo C, Tuohy KM (2018). Host: microbiome co-metabolic processing of dietary polyphenols–an acute, single blinded, cross-over study with different doses of apple polyphenols in healthy subjects. Food Res Int.

[17] Caporaso JG, Lauber CL, Walters WA, Berg-Lyons D, Lozupone CA, Turnbaugh PJ (2011). Global patterns of 16S rRNA diversity at a depth of millions of sequences per sample. Proc Natl Acad Sci.

[18] White TJ, Bruns T, Lee S, Taylor J (1990). Amplification and direct sequencing of fungal ribosomal RNA genes for phylogenetics. PCR Protoc Guide Methods Appl.

[19] Köberl M, Müller H, Ramadan EM, Berg G (2011) Desert farming benefits from microbial potential in arid soils and promotes diversity and plant health. PLoS One 6:e24452 10.1371/journal.pone.0024452 PMC3166316 21912695

[20] Martin M (2011). Cutadapt removes adapter sequences from high-throughput sequencing reads. EMBnet J.

[21] Callahan BJ, McMurdie PJ, Rosen MJ, Han AW, Johnson AJA, Holmes SP (2016). DADA2: high-resolution sample inference from Illumina amplicon data. Nat Methods.

[22] Bolyen E, Rideout JR, Dillon MR, Bokulich NA, Abnet CC, Al-Ghalith GA (2019). Reproducible, interactive, scalable and extensible microbiome data science using QIIME 2. Nat Biotechnol.

[23] Pruesse E, Quast C, Knittel K, Fuchs BM, Ludwig W, Peplies J (2007). SILVA: a comprehensive online resource for quality checked and aligned ribosomal RNA sequence data compatible with ARB. Nucleic Acids Res.

[24] Rognes T, Flouri T, Nichols B, Quince C, Mahé F (2016). VSEARCH: a versatile open source tool for metagenomics. PeerJ.

[25] Abarenkov K, Henrik Nilsson R, Larsson K, Alexander IJ, Eberhardt U, Erland S (2010). The UNITE database for molecular identification of fungi–recent updates and future perspectives. New Phytol.

[26] Allaire J (2012). RStudio: integrated development environment for R. Boston MA.

[27] Chong J, Liu P, Zhou G, Xia J (2020). Using MicrobiomeAnalyst for comprehensive statistical, functional, and meta-analysis of microbiome data. Nat Protoc.

[28] McMurdie PJ, Holmes S (2013) phyloseq: an R package for reproducible interactive analysis and graphics of microbiome census data. PloS One 8: e61217 10.1371/journal.pone.0061217 PMC3632530 23630581

[29] Segata N, Izard J, Waldron L, Gevers D, Miropolsky L, Garrett WS (2011). Metagenomic biomarker discovery and explanation. Genome Biol.

[30] Leff JW, Fierer N (2013). Bacterial communities associated with the surfaces of fresh fruits and vegetables. PLoS ONE.

[31] Muñoz-Arenas LC, Fusaro C, Hernández-Guzmán M, Dendooven L, Estrada-Torres A, Navarro-Noya YE (2020). Soil microbial diversity drops with land-use change in a high mountain temperate forest: a metagenomics survey. Environ Microbiol Rep.

[32] Tsiafouli MA, Thébault E, Sgardelis SP, De Ruiter PC, Van Der Putten WH, Birkhofer K (2015). Intensive agriculture reduces soil biodiversity across Europe. Glob Change Biol.

[33] Massoni J, Bortfeld-Miller M, Widmer A, Vorholt JA (2021) Capacity of soil bacteria to reach the phyllosphere and convergence of floral communities despite soil microbiota variation. Proc Natl Acad Sci 118:e2100150118 10.1073/pnas.2100150118 PMC8521660 34620708

[34] Zarraonaindia I, Owens SM, Weisenhorn P, West K, Hampton-Marcell J, Lax S (2015). The soil microbiome influences grapevine-associated microbiota. MBio.

[35] Abdelfattah A, Freilich S, Bartuv R, Zhimo VY, Kumar A, Biasi A (2021). Global analysis of the apple fruit microbiome: are all apples the same?. Environ Microbiol.

[36] del Carmen PM, Franquès J, Araque I, Reguant C, Bordons A (2016). Bacterial diversity of Grenache and Carignan grape surface from different vineyards at Priorat wine region (Catalonia, Spain). Int J Food Microbiol.

[37] Mezzasalma V, Sandionigi A, Bruni I, Bruno A, Lovicu G, Casiraghi M (2017). Grape microbiome as a reliable and persistent signature of field origin and environmental conditions in Cannonau wine production. PLoS ONE.

[38] Maheswari P, Raja P, Apolo-Apolo OE, Pérez-Ruiz M (2021). Intelligent fruit yield estimation for orchards using deep learning based semantic segmentation techniques—a review. Front Plant Sci.

[39] Prohens J, Nuez F (2001). The tamarillo (Cyphomandra betacea) a review of a promising small fruit crop. Small Fruits Rev.

[40] Zalapa JE, Staub J, McCreight J (2006). Generation means analysis of plant architectural traits and fruit yield in melon. Plant Breed.

[41] Cordovez V, Dini-Andreote F, Carrión VJ, Raaijmakers JM (2019). Ecology and evolution of plant microbiomes. Annu Rev Microbiol.

[42] Lupatini M, Korthals GW, de Hollander M, Janssens TK, Kuramae EE (2017). Soil microbiome is more heterogeneous in organic than in conventional farming system. Front Microbiol.

[43] González-Rodríguez MI, Nurminen N, Kummola L, Laitinen OH, Oikarinen S, Parajuli A (2022). Effect of inactivated nature-derived microbial composition on mouse immune system. Immun Inflamm Dis.

[44] Wankhade UD, Zhong Y, Lazarenko OP, Chintapalli SV, Piccolo BD, Chen J-R (2019). Sex-specific changes in gut microbiome composition following blueberry consumption in C57BL/6J mice. Nutrients.

[45] Flandroy L, Poutahidis T, Berg G, Clarke G, Dao M-C, Decaestecker E (2018). The impact of human activities and lifestyles on the interlinked microbiota and health of humans and of ecosystems. Sci Total Environ.

[46] Brooke JS (2012). Stenotrophomonas maltophilia: an emerging global opportunistic pathogen. Clin Microbiol Rev.

[47] Hu M, Li C, Xue Y, Hu A, Chen S, Chen Y (2021). Isolation, characterization, and genomic investigation of a phytopathogenic strain of Stenotrophomonas maltophilia. Phytopathology.

[48] Ling L, Jiao Z, Ma W, Zhao J, Feng J, Zhang X (2019). Preliminary report on the study of postharvest fruit rot bacteria and yeasts in Lanzhou Lily (Lilium davidii var. unicolor) in China. J Phytopathol.

[49] Pusey P, Stockwell V, Reardon C, Smits T, Duffy B (2011). Antibiosis activity of Pantoea agglomerans biocontrol strain E325 against Erwinia amylovora on apple flower stigmas. Phytopathology.

[50] Cruz AT, Cazacu AC, Allen CH (2007). Pantoea agglomerans, a plant pathogen causing human disease. J Clin Microbiol.

[51] Berger CN, Sodha SV, Shaw RK, Griffin PM, Pink D, Hand P (2010). Fresh fruit and vegetables as vehicles for the transmission of human pathogens. Environ Microbiol.

[52] Cho G-S, Stein M, Fiedler G, Igbinosa EO, Koll LP, Brinks E et al (2021) Polyphasic study of antibiotic-resistant enterobacteria isolated from fresh produce in Germany and description of Enterobacter vonholyi sp. nov. isolated from marjoram and Enterobacter dykesii sp. nov. isolated from mung bean sprout. Syst Appl Microbiol 44:126174 10.1016/j.syapm.2020.126174 33370657

[53] Lukša J, Vepštaitė-Monstavičė I, Yurchenko V, Serva S, Servienė E (2018). High content analysis of sea buckthorn, black chokeberry, red and white currants microbiota–a pilot study. Food Res Int.

[54] Choi O, Lee Y, Kang B, Kim S, Bae J, Kim J (2020). Bacterial shoot blight of sweet crab apple caused by Pseudomonas viridiflava. For Pathol.

[55] Lipps SM, Samac DA (2021). Pseudomonas viridiflava: an internal outsider of the Pseudomonas syringae species complex. Mol Plant Pathol.

[56] Kwon J-H, Cheon M-G, Choi O, Kim J (2011). First report of Botrytis cinerea as a postharvest pathogen of blueberry in Korea. Mycobiology.

[57] Saito S, Michailides T, Xiao C-L (2016). Fungicide resistance profiling in Botrytis cinerea populations from blueberry in California and Washington and their impact on control of gray mold. Plant Dis.

[58] Del Frari G, Cabral A, Nascimento T, Boavida Ferreira R, Oliveira H (2019). Epicoccum layuense a potential biological control agent of esca-associated fungi in grapevine. PLoS ONE.

[59] Wassermann B, Cernava T, Müller H, Berg C, Berg G (2019). Seeds of native alpine plants host unique microbial communities embedded in cross-kingdom networks. Microbiome.

[60] Droby S, Wisniewski M (2018). The fruit microbiome: a new frontier for postharvest biocontrol and postharvest biology. Postharvest Biol Technol.

[61] Kusstatscher P, Cernava T, Abdelfattah A, Gokul J, Korsten L, Berg G (2020) Microbiome approaches provide the key to biologically control postharvest pathogens and storability of fruits and vegetables. FEMS Microbiol Ecol 96:fiaa119 10.1093/femsec/fiaa119 32542314

[62] Liu NT, Lefcourt AM, Nou X, Shelton DR, Zhang G, Lo YM (2013). Native microflora in fresh-cut produce processing plants and their potentials for biofilm formation. J Food Prot.

[63] Liu NT, Nou X, Lefcourt AM, Shelton DR, Lo YM (2014). Dual-species biofilm formation by Escherichia coli O157: H7 and environmental bacteria isolated from fresh-cut processing facilities. Int J Food Microbiol.

[64] Busse H-J, Denner EB, Buczolits S, Salkinoja-Salonen M, Bennasar A, Kämpfer P (2003). Sphingomonas aurantiaca sp. nov., Sphingomonas aerolata sp. nov. and Sphingomonas faeni sp. nov., air-and dustborne and Antarctic, orange-pigmented, psychrotolerant bacteria, and emended description of the genus Sphingomonas. Int J Syst Evol Microbiol.

[65] Mageswari A, Subramanian P, Ravindran V, Yesodharan S, Bagavan A, Rahuman AA (2015). Synthesis and larvicidal activity of low-temperature stable silver nanoparticles from psychrotolerant Pseudomonas mandelii. Environ Sci Pollut Res.

[66] Saleh-Lakha S, Shannon KE, Henderson SL, Goyer C, Trevors JT, Zebarth BJ (2009). Effect of pH and temperature on denitrification gene expression and activity in Pseudomonas mandelii. Appl Environ Microbiol.

[67] Kusstatscher P, Adam E, Wicaksono WA, Bernhart M, Olimi E, Müller H et al (2021) Microbiome-assisted breeding to understand cultivar-dependent assembly in Cucurbita pepo. Front Plant Sci 12 10.3389/fpls.2021.642027 PMC8063107 33897731

[68] Morales Moreira ZP, Helgason BL, Germida JJ (2021). Environment has a stronger effect than host plant genotype in shaping spring Brassica napus seed microbiomes. Phytobiomes J.

[69] Sun D, Qu J, Huang Y, Lu J, Yin L (2021). Analysis of microbial community diversity of muscadine grape skins. Food Res Int.

