# Supplementary material for: Impact of Cultivation and Origin on the Fruit Microbiome of Apples and Blueberries and Implications for the Exposome
Source: Microb Ecol. 2022 Dec 21;86(2):973–84. doi: 10.1007/s00248-022-02157-8 (PMC10335955; doi:10.1007/s00248-022-02157-8)
Supplement: Supplementary file 1 — Supplementary file1 (DOCX 1630 KB) [file 248_2022_2157_MOESM1_ESM.docx]

**Supplementary Material**

**Impact of cultivation and origin on the fruit microbiome of apples and blueberries and implications for the exposome**

Wisnu Adi Wicaksono^1#^, Aisa Buko^1^, Peter Kusstatscher^1^, Tomislav Cernava^1^, Aki Sinkkonen^2^, Olli H Laitinen^3^, Suvi M. Virtanen^4,5,6,7^, Heikki Hyöty^3,8^, Gabriele Berg^1,9,10#^

^1^Institute of Environmental Biotechnology, Graz University of Technology, Graz, Austria

^2^ Natural Resources Institute Finland Luke, Turku, Finland

^3^ Faculty of Medicine and Health Technology, Tampere University, Tampere, Finland

^4^ Health and Well-Being Promotion Unit, Finnish Institute for Health and Welfare, Helsinki, Finland

^5^ Faculty of Social Sciences, Unit of Health Sciences, Tampere University, Tampere, Finland ^6^ Research, Development and Innovation Centre, Tampere University Hospital, Tampere, Finland

^7^ Center for Child Health Research, Tampere University and Tampere University Hospital, Tampere, Finland

^8^ Fimlab Laboratories, Pirkanmaa Hospital District, Tampere, Finland

^9^ Leibniz Institute for Agricultural Engineering and Bioeconomy (ATB), Potsdam, Germany

^10^ Institute for Biochemistry and Biology, University of Potsdam, Potsdam, Germany

^#^Corresponding author:

Wisnu Adi Wicaksono

Email: [wisnu.wicaksono@tugraz.at](mailto:wisnu.wicaksono@tugraz.at)

Gabriele Berg

Email: gabriele.berg@tugraz.at

**Supplementary Materials**

**Supplementary Table S1. Detailed sample description for apples and blueberries obtained in Austria and Finland.**

| Sample ID | Fruit | Growing system | Country | Location | Species/Cultivar |
| --- | --- | --- | --- | --- | --- |
| AP1 | Apple | Natural | Austria | Graz_1 | N. A |
| AP2 | Apple | Natural | Austria | Graz_1 | N. A |
| AP3 | Apple | Natural | Austria | Graz_1 | N. A |
| AP4 | Apple | Natural | Austria | Graz_2 | N. A |
| AP5 | Apple | Natural | Austria | Graz_2 | N. A |
| AP6 | Apple | Natural | Austria | Graz_2 | N. A |
| AP7 | Apple | Natural | Austria | Graz_3 | N. A |
| AP8 | Apple | Natural | Austria | Graz_3 | N. A |
| AP9 | Apple | Natural | Austria | Graz_3 | N. A |
| AP10 | Apple | Natural | Austria | Graz_4 | N. A |
| AP11 | Apple | Natural | Austria | Graz_4 | N. A |
| AP12 | Apple | Natural | Austria | Graz_4 | N. A |
| AP13 | Apple | Natural | Austria | Graz_5 | N. A |
| AP14 | Apple | Natural | Austria | Graz_5 | N. A |
| AP15 | Apple | Natural | Austria | Graz_5 | N. A |
| AP16 | Apple | Natural | Austria | Graz_6 | N. A |
| AP17 | Apple | Natural | Austria | Graz_6 | N. A |
| AP18 | Apple | Natural | Austria | Graz_6 | N. A |
| AP19 | Apple | Natural | Austria | Weiz_1 | Summered |
| AP20 | Apple | Natural | Austria | Weiz_1 | Summered |
| AP21 | Apple | Natural | Austria | Weiz_1 | Summered |
| AP22 | Apple | Natural | Austria | Weiz_2 | Mantet |
| AP23 | Apple | Natural | Austria | Weiz_2 | Mantet |
| AP24 | Apple | Natural | Austria | Weiz_2 | Mantet |
| AP25 | Apple | Natural | Austria | Weiz_2 | Gloster |
| AP26 | Apple | Natural | Austria | Weiz_3 | Gloster |
| AP27 | Apple | Natural | Austria | Weiz_3 | Gloster |
| AP28 | Apple | Natural | Austria | Murtal_1 | N. A |
| AP29 | Apple | Natural | Austria | Murtal_1 | N. A |
| AP30 | Apple | Natural | Austria | Murtal_1 | N. A |
| AP31 | Apple | Natural | Austria | Murtal_2 | N. A |
| AP32 | Apple | Natural | Austria | Murtal_2 | N. A |
| AP33 | Apple | Natural | Austria | Murtal_2 | N. A |
| AP34 | Apple | Natural | Austria | Murtal_3 | N. A |
| AP35 | Apple | Natural | Austria | Murtal_3 | N. A |
| AP36 | Apple | Natural | Austria | Murtal_3 | N. A |
| AP37 | Apple | Natural | Austria | Murtal_4 | N. A |
| AP38 | Apple | Natural | Austria | Murtal_4 | N. A |
| AP39 | Apple | Natural | Austria | Murtal_4 | N. A |
| AP40 | Apple | Natural | Austria | Südoststeiermark_1 | Grafensteiner |
| AP41 | Apple | Natural | Austria | Südoststeiermark_1 | Grafensteiner |
| AP42 | Apple | Natural | Austria | Südoststeiermark_1 | Grafensteiner |
| AP43 | Apple | Natural | Austria | Güssing_1 | Grafensteiner |
| AP44 | Apple | Natural | Austria | Güssing_1 | Grafensteiner |
| AP45 | Apple | Natural | Austria | Güssing_1 | Grafensteiner |
| AP46 | Apple | Natural | Finland | Hirvensalmi_1 | Valkea kuulas |
| AP47 | Apple | Natural | Finland | Hirvensalmi_1 | Valkea kuulas |
| AP48 | Apple | Natural | Finland | Hirvensalmi_1 | Valkea kuulas |
| AP49 | Apple | Natural | Finland | Hirvensalmi_2 | Petteri |
| AP50 | Apple | Natural | Finland | Hirvensalmi_2 | Petteri |
| AP51 | Apple | Natural | Finland | Hirvensalmi_2 | Petteri |
| AP52 | Apple | Natural | Finland | Hirvensalmi_3 | Sarso |
| AP53 | Apple | Natural | Finland | Hirvensalmi_3 | Sarso |
| AP54 | Apple | Natural | Finland | Hirvensalmi_3 | Sarso |
| AP55 | Apple | Natural | Finland | Vantaa_1 | Huvitus |
| AP56 | Apple | Natural | Finland | Vantaa_1 | Huvitus |
| AP57 | Apple | Natural | Finland | Vantaa_1 | Huvitus |
| AP58 | Apple | Natural | Finland | Vantaa_2 | N. A |
| AP59 | Apple | Natural | Finland | Vantaa_2 | N. A |
| AP60 | Apple | Natural | Finland | Vantaa_2 | N. A |
| AP61 | Apple | Horticultural | Finland | Store F1 | Pirja |
| AP62 | Apple | Horticultural | Finland | Store F1 | Pirja |
| AP63 | Apple | Horticultural | Finland | Store F1 | Pirja |
| AP64 | Apple | Horticultural | Finland | Store F2 | Royal Gala |
| AP65 | Apple | Horticultural | Finland | Store F2 | Royal Gala |
| AP66 | Apple | Horticultural | Finland | Store F2 | Royal Gala |
| AP67 | Apple | Horticultural | Finland | Store F2 | Royal Gala |
| AP68 | Apple | Horticultural | Finland | Store F2 | Royal Gala |
| AP69 | Apple | Horticultural | Finland | Store F2 | Royal Gala |
| AP70 | Apple | Horticultural | Finland | Store F2 | Golden delicious |
| AP71 | Apple | Horticultural | Finland | Store F2 | Golden delicious |
| AP72 | Apple | Horticultural | Finland | Store F2 | Golden delicious |
| AP73 | Apple | Horticultural | Finland | Store F2 | Granny smith |
| AP74 | Apple | Horticultural | Finland | Store F2 | Granny smith |
| AP75 | Apple | Horticultural | Finland | Store F2 | Granny smith |
| AP76 | Apple | Horticultural | Finland | Store F3 | Red melba |
| AP77 | Apple | Horticultural | Finland | Store F3 | Red melba |
| AP78 | Apple | Horticultural | Finland | Store F3 | Red melba |
| AP79 | Apple | Horticultural | Finland | Store F3 | Ida red |
| AP80 | Apple | Horticultural | Finland | Store F3 | Ida red |
| AP81 | Apple | Horticultural | Finland | Store F3 | Ida red |
| AP82 | Apple | Horticultural | Austria | Store_A1 | Gala |
| AP83 | Apple | Horticultural | Austria | Store_A1 | Gala |
| AP84 | Apple | Horticultural | Austria | Store_A1 | Gala |
| AP85 | Apple | Horticultural | Austria | Store_A1 | Golden delicious |
| AP86 | Apple | Horticultural | Austria | Store_A1 | Golden delicious |
| AP87 | Apple | Horticultural | Austria | Store_A1 | Golden delicious |
| AP88 | Apple | Horticultural | Austria | Store_A1 | Summer |
| AP89 | Apple | Horticultural | Austria | Store_A1 | Summer |
| AP90 | Apple | Horticultural | Austria | Store_A1 | Summer |
| AP91 | Apple | Horticultural | Austria | Store_A1 | Red Jonaprinz |
| AP92 | Apple | Horticultural | Austria | Store_A1 | Red Jonaprinz |
| AP93 | Apple | Horticultural | Austria | Store_A1 | Red Jonaprinz |
| AP94 | Apple | Horticultural | Austria | Store_A2 | Gala |
| AP95 | Apple | Horticultural | Austria | Store_A2 | Gala |
| AP96 | Apple | Horticultural | Austria | Store_A2 | Gala |
| AP97 | Apple | Horticultural | Austria | Store_A2 | Golden delicious |
| AP98 | Apple | Horticultural | Austria | Store_A2 | Golden delicious |
| AP99 | Apple | Horticultural | Austria | Store_A2 | Golden delicious |
| AP100 | Apple | Horticultural | Austria | Store_A3 | Gala |
| AP101 | Apple | Horticultural | Austria | Store_A3 | Gala |
| AP102 | Apple | Horticultural | Austria | Store_A3 | Gala |
| AP103 | Apple | Horticultural | Austria | Store_A3 | Ida red |
| AP104 | Apple | Horticultural | Austria | Store_A3 | Ida red |
| AP105 | Apple | Horticultural | Austria | Store_A3 | Ida red |
| AP106 | Apple | Horticultural | Austria | Store_A3 | Golden delicious |
| AP107 | Apple | Horticultural | Austria | Store_A3 | Golden delicious |
| AP108 | Apple | Horticultural | Austria | Store_A3 | Golden delicious |
| BL1 | Blueberry | Horticultural | Austria | Store_A1 | *V. corymbosum* |
| BL2 | Blueberry | Horticultural | Austria | Store_A1 | *V. corymbosum* |
| BL3 | Blueberry | Horticultural | Austria | Store_A1 | *V. corymbosum* |
| BL4 | Blueberry | Horticultural | Austria | Store_A1 | *V. corymbosum* |
| BL5 | Blueberry | Horticultural | Austria | Store_A1 | *V. corymbosum* |
| BL6 | Blueberry | Horticultural | Austria | Store_A1 | *V. corymbosum* |
| BL7 | Blueberry | Horticultural | Austria | Store_A2 | *V. corymbosum* |
| BL8 | Blueberry | Horticultural | Austria | Store_A2 | *V. corymbosum* |
| BL9 | Blueberry | Horticultural | Austria | Store_A2 | *V. corymbosum* |
| BL10 | Blueberry | Horticultural | Austria | Store_A2 | *V. corymbosum* |
| BL11 | Blueberry | Horticultural | Austria | Store_A2 | *V. corymbosum* |
| BL12 | Blueberry | Horticultural | Austria | Store_A2 | *V. corymbosum* |
| BL13 | Blueberry | Horticultural | Austria | Store_A3 | *V. corymbosum* |
| BL14 | Blueberry | Horticultural | Austria | Store_A3 | *V. corymbosum* |
| BL15 | Blueberry | Horticultural | Austria | Store_A3 | *V. corymbosum* |
| BL16 | Blueberry | Horticultural | Austria | Store_A3 | *V. corymbosum* |
| BL17 | Blueberry | Horticultural | Austria | Store_A3 | *V. corymbosum* |
| BL18 | Blueberry | Horticultural | Austria | Store_A3 | *V. corymbosum* |
| BL19 | Blueberry | Horticultural | Austria | Store_A1 | *V. corymbosum* |
| BL20 | Blueberry | Horticultural | Austria | Store_A1 | *V. corymbosum* |
| BL21 | Blueberry | Horticultural | Austria | Store_A1 | *V. corymbosum* |
| BL22 | Blueberry | Horticultural | Austria | Store_A1 | *V. corymbosum* |
| BL23 | Blueberry | Horticultural | Austria | Store_A1 | *V. corymbosum* |
| BL24 | Blueberry | Horticultural | Austria | Store_A1 | *V. corymbosum* |
| BL25 | Blueberry | Horticultural | Austria | Store_A2 | *V. corymbosum* |
| BL26 | Blueberry | Horticultural | Austria | Store_A2 | *V. corymbosum* |
| BL27 | Blueberry | Horticultural | Austria | Store_A2 | *V. corymbosum* |
| BL28 | Blueberry | Horticultural | Austria | Store_A2 | *V. corymbosum* |
| BL29 | Blueberry | Horticultural | Austria | Store_A2 | *V. corymbosum* |
| BL30 | Blueberry | Horticultural | Austria | Store_A2 | *V. corymbosum* |
| BL31 | Blueberry | Horticultural | Austria | Store_A3 | *V. corymbosum* |
| BL32 | Blueberry | Horticultural | Austria | Store_A3 | *V. corymbosum* |
| BL33 | Blueberry | Horticultural | Austria | Store_A3 | *V. corymbosum* |
| BL34 | Blueberry | Horticultural | Austria | Store_A3 | *V. corymbosum* |
| BL35 | Blueberry | Horticultural | Austria | Store_A3 | *V. corymbosum* |
| BL36 | Blueberry | Horticultural | Austria | Store_A3 | *V. corymbosum* |
| BL37 | Blueberry | Natural | Austria | Murtal_5 | *V. myrtillus* |
| BL38 | Blueberry | Natural | Austria | Murtal_5 | *V. myrtillus* |
| BL39 | Blueberry | Natural | Austria | Murtal_5 | *V. myrtillus* |
| BL40 | Blueberry | Natural | Austria | Murtal_5 | *V. myrtillus* |
| BL41 | Blueberry | Natural | Austria | Murtal_1 | *V. corymbosum* |
| BL42 | Blueberry | Natural | Austria | Murtal_1 | *V. corymbosum* |
| BL43 | Blueberry | Natural | Austria | Murtal_1 | *V. corymbosum* |
| BL44 | Blueberry | Natural | Austria | Murtal_1 | *V. corymbosum* |
| BL45 | Blueberry | Natural | Austria | Südoststeiermark_2 | *V. myrtillus* |
| BL46 | Blueberry | Natural | Austria | Südoststeiermark_2 | *V. myrtillus* |
| BL47 | Blueberry | Natural | Austria | Südoststeiermark_2 | *V. myrtillus* |
| BL48 | Blueberry | Natural | Austria | Südoststeiermark_2 | *V. myrtillus* |
| BL49 | Blueberry | Natural | Austria | Graz_7 | *V. myrtillus* |
| BL50 | Blueberry | Natural | Austria | Graz_7 | *V. myrtillus* |
| BL51 | Blueberry | Natural | Austria | Graz_7 | *V. myrtillus* |
| BL52 | Blueberry | Natural | Austria | Graz_7 | *V. myrtillus* |
| BL53 | Blueberry | Horticultural | Finland | Store F2 | *V. corymbosum* |
| BL54 | Blueberry | Horticultural | Finland | Store F2 | *V. corymbosum* |
| BL55 | Blueberry | Horticultural | Finland | Store F2 | *V. corymbosum* |
| BL56 | Blueberry | Horticultural | Finland | Store F2 | *V. corymbosum* |
| BL57 | Blueberry | Horticultural | Finland | Store F2 | *V. corymbosum* |
| BL58 | Blueberry | Horticultural | Finland | Store F2 | *V. corymbosum* |
| BL59 | Blueberry | Horticultural | Finland | Store F3 | *V. corymbosum* |
| BL60 | Blueberry | Horticultural | Finland | Store F3 | *V. corymbosum* |
| BL61 | Blueberry | Horticultural | Finland | Store F3 | *V. corymbosum* |
| BL62 | Blueberry | Horticultural | Finland | Store F3 | *V. corymbosum* |
| BL63 | Blueberry | Horticultural | Finland | Store F3 | *V. corymbosum* |
| BL64 | Blueberry | Horticultural | Finland | Store F3 | *V. corymbosum* |
| BL65 | Blueberry | Horticultural | Finland | Store F3 | *V. corymbosum* |
| BL66 | Blueberry | Horticultural | Finland | Store F3 | *V. corymbosum* |
| BL67 | Blueberry | Horticultural | Finland | Store F3 | *V. corymbosum* |
| BL68 | Blueberry | Horticultural | Finland | Store F3 | *V. corymbosum* |
| BL69 | Blueberry | Horticultural | Finland | Store F3 | *V. corymbosum* |
| BL70 | Blueberry | Horticultural | Finland | Store F3 | *V. corymbosum* |
| BL71 | Blueberry | Horticultural | Finland | Store F2 | *V. corymbosum* |
| BL72 | Blueberry | Horticultural | Finland | Store F2 | *V. corymbosum* |
| BL73 | Blueberry | Horticultural | Finland | Store F2 | *V. corymbosum* |
| BL74 | Blueberry | Horticultural | Finland | Store F2 | *V. corymbosum* |
| BL75 | Blueberry | Horticultural | Finland | Store F2 | *V. corymbosum* |
| BL76 | Blueberry | Horticultural | Finland | Store F2 | *V. corymbosum* |
| BL77 | Blueberry | Natural | Austria | Deutschlandsberg_1 | *V. corymbosum* |
| BL78 | Blueberry | Natural | Austria | Deutschlandsberg_1 | *V. corymbosum* |
| BL79 | Blueberry | Natural | Austria | Deutschlandsberg_1 | *V. corymbosum* |
| BL80 | Blueberry | Natural | Austria | Deutschlandsberg_1 | *V. corymbosum* |
| BL81 | Blueberry | Natural | Austria | Deutschlandsberg_2 | *V. corymbosum* |
| BL82 | Blueberry | Natural | Austria | Deutschlandsberg_2 | *V. corymbosum* |
| BL83 | Blueberry | Natural | Austria | Deutschlandsberg_2 | *V. corymbosum* |
| BL84 | Blueberry | Natural | Austria | Deutschlandsberg_2 | *V. corymbosum* |
| BL89 | Blueberry | Natural | Finland | Hietasaari_1 | *V. myrtillus* |
| BL90 | Blueberry | Natural | Finland | Hietasaari_1 | *V. myrtillus* |
| BL91 | Blueberry | Natural | Finland | Hietasaari_1 | *V. myrtillus* |
| BL92 | Blueberry | Natural | Finland | Hietasaari_1 | *V. myrtillus* |
| BL93 | Blueberry | Natural | Finland | Korpisaari_1 | *V. myrtillus* |
| BL94 | Blueberry | Natural | Finland | Korpisaari_1 | *V. myrtillus* |
| BL95 | Blueberry | Natural | Finland | Korpisaari_1 | *V. myrtillus* |
| BL96 | Blueberry | Natural | Finland | Korpisaari_1 | *V. myrtillus* |
| BL97 | Blueberry | Natural | Finland | Mainrenniemi | *V. myrtillus* |
| BL98 | Blueberry | Natural | Finland | Mainrenniemi | *V. myrtillus* |
| BL99 | Blueberry | Natural | Finland | Mainrenniemi | *V. myrtillus* |
| BL100 | Blueberry | Natural | Finland | Mainrenniemi | *V. myrtillus* |
| BL101 | Blueberry | Natural | Finland | Ryökäsvesi_1 | *V. myrtillus* |
| BL102 | Blueberry | Natural | Finland | Ryökäsvesi_1 | *V. myrtillus* |
| BL103 | Blueberry | Natural | Finland | Ryökäsvesi_1 | *V. myrtillus* |
| BL104 | Blueberry | Natural | Finland | Ryökäsvesi_1 | *V. myrtillus* |

**Supplementary Table S2. Pairwise comparisons of community compositions of apples and blueberries from different growing systems and countries of origin.**

|  | Fruit | Pairwise comparison | *P*_adj_ |
| --- | --- | --- | --- |
| Bacteria | Apple | Naturally grown Austria vs Horticultural  Austria | 0.002* |
|  |  | Naturally grown Finland vs Horticultural Finland | 0.079 |
|  |  | Naturally grown Finland vs Naturally grown Austria | 0.092 |
|  |  | Horticultural Austria vs Horticultural Finland | 0.002* |
|  |  | Horticultural Austria vs Naturally grown Finland | 0.002* |
|  |  | Naturally grown Austria vs Horticultural Finland | 0.010* |
|  | Blueberry | Naturally grown Austria vs Horticultural Austria | 0.002* |
|  |  | Naturally grown Finland vs Horticultural Finland | 0.002* |
|  |  | Naturally grown Finland vs Naturally grown Austria | 0.011* |
|  |  | Horticultural Austria vs Horticultural Finland | 0.002* |
|  |  | Horticultural Austria vs Naturally grown Finland | 0.002* |
|  |  | Naturally grown Austria vs Horticultural Finland | 0.011* |
| Fungi | Apple | Naturally grown Austria vs Horticultural Austria | 0.002* |
|  |  | Naturally grown Finland vs Horticultural Finland | 0.010* |
|  |  | Naturally grown Finland vs Naturally grown Austria | 0.003* |
|  |  | Horticultural Austria vs Horticultural Finland | 0.004* |
|  |  | Horticultural Austria vs Naturally grown Finland | 0.002* |
|  |  | Naturally grown Austria vs Horticultural Finland | 0.002* |
|  | Blueberry | Naturally grown Austria vs Horticultural Austria | 0.002* |
|  |  | Naturally grown Finland vs Horticultural Finland | 0.002* |
|  |  | Naturally grown Finland vs Naturally grown Austria | 0.006* |
|  |  | Horticultural Austria vs Horticultural Finland | 0.002* |
|  |  | Horticultural Austria vs Naturally grown Finland | 0.002* |
|  |  | Naturally grown Austria vs Horticultural Finland | 0.002* |

**Supplementary Table S3. Identification of specific bacterial ASVs based on BLAST analysis.**

| Dataset | ASV ID | *P* value | LDA  score | NCBI BLAST match | Accession no. | Relative abundance (%) | |
| --- | --- | --- | --- | --- | --- | --- | --- |
|  |  |  |  |  |  | Horticultural | Naturally grown |
| Apple | *Pseudomonas* ASV1 | <0.001 | 2.1 | *Pseudomonas mandeli* | OK135852.1 | 51.9 | 27.8 |
|  | *Ralstonia* ASV2 | <0.001 | 1.5 | *Ralstonia insidiosa* | MT394016.1 | 8.7 | 2.7 |
|  | *Stenotrophomonas* ASV3 | <0.001 | 1.0 | *Stenotrophomonas maltophilia* | MT533812.1 | 4.2 | 2.3 |
|  | *Pseudomonas* ASV4 | 0.007 | -1.1 | *Pseudomonas viridiflava* | MT605451.1 | 3.4 | 5.8 |
|  | *Pseudomonas* ASV5 | 0.012 | -1.2 | *Pseudomonas gingeri* | KX817286.1 | 2.5 | 5.0 |
|  | *Pantoea* ASV7 | <0.001 | -1.2 | *Pantoea agglomerans* | MT635441.1 | 1.5 | 4.4 |
|  | Unc. *Enterobacteriaceae* ASV8 | <0.001 | -1.6 | *Tatumella ptyseos* | MK720714.1 | 0.4 | 8.1 |
| Blueberry | Unc. *Enterobacteriaceae* ASV8 | <0.001 | 1.9 | *Tatumella ptyseos* | MK720714.1 | 7.8 | 0.8 |
|  | Unc. *Enterobacteriaceae* ASV9 | 0.041 | 1.6 | *Serratia sp* | MT626726.1 | 7.8 | 3.7 |
|  | *Ralstonia* ASV2 | <0.001 | 1.2 | *Ralstonia insidiosa* | MT394016.1 | 3.3 | 1.9 |
|  | *Sphingomonas* ASV10 | <0.001 | 1.1 | *Sphingomonas aerolata* | LR722768.1 | 3.0 | 1.8 |
|  | Unc. *Enterobacteriaceae* ASV11 | 0.004 | -1.1 | *Enterobacter hormaechei* | CP044335.1 | 0.5 | 1.6 |
|  | *Komagataeibacter* ASV12 | <0.001 | -1.1 | *Komagataeibacter intermedius* | MK099873.1 | 0.2 | 1.4 |
|  | Unc. *Burkholderiaceae* ASV13 | 0.024 | -1.3 | *Robbsia sp.* | MT012253.1 | 0.9 | 2.6 |
|  | *Dyella* ASV14 | 0.029 | -1.4 | *Dyella japonica* | KC241898.1 | 0.8 | 3.3 |
|  | *Gluconobacter* ASV15 | <0.001 | -1.8 | *Gluconobacter cerinus* | MN367105.1 | 1.1 | 7.6 |

**Supplementary Table S4. Identification of specific fungal ASVs based on BLAST analysis.**

| Dataset | ASV ID | Pvalues | LDA score | NCBI BLAST match | Accession no. | Relative abundance (%) | |
| --- | --- | --- | --- | --- | --- | --- | --- |
|  |  |  |  |  |  | Horticultural | Naturally grown |
| Apple | *Cladosporium* ASV1 | 0.016 | 1.1 | *Cladosporium cladosporioides* | MT609901.1 | 16.6 | 11.8 |
| Blueberry | Unc. *Sclerotiniaceae* ASV2 | <0.001 | 1.3 | *Botrytis cinerea* | MT573470.1 | 10.3 | 1.4 |
|  | *Cladosporium* ASV3 | <0.001 | 1.3 | *Cladosporium allicinum* | MT573471.1 | 12.5 | 3.7 |
|  | Unc. *Didymellaceae* ASV4 | <0.001 | 1.1 | *Epicoccum layuense* | MT573479.1 | 5.4 | 0.6 |
|  | *Cladosporium* ASV1 | 0.003 | 1.1 | *Cladosporium cladosporioides* | MT609901.1 | 14.3 | 9.3 |

**
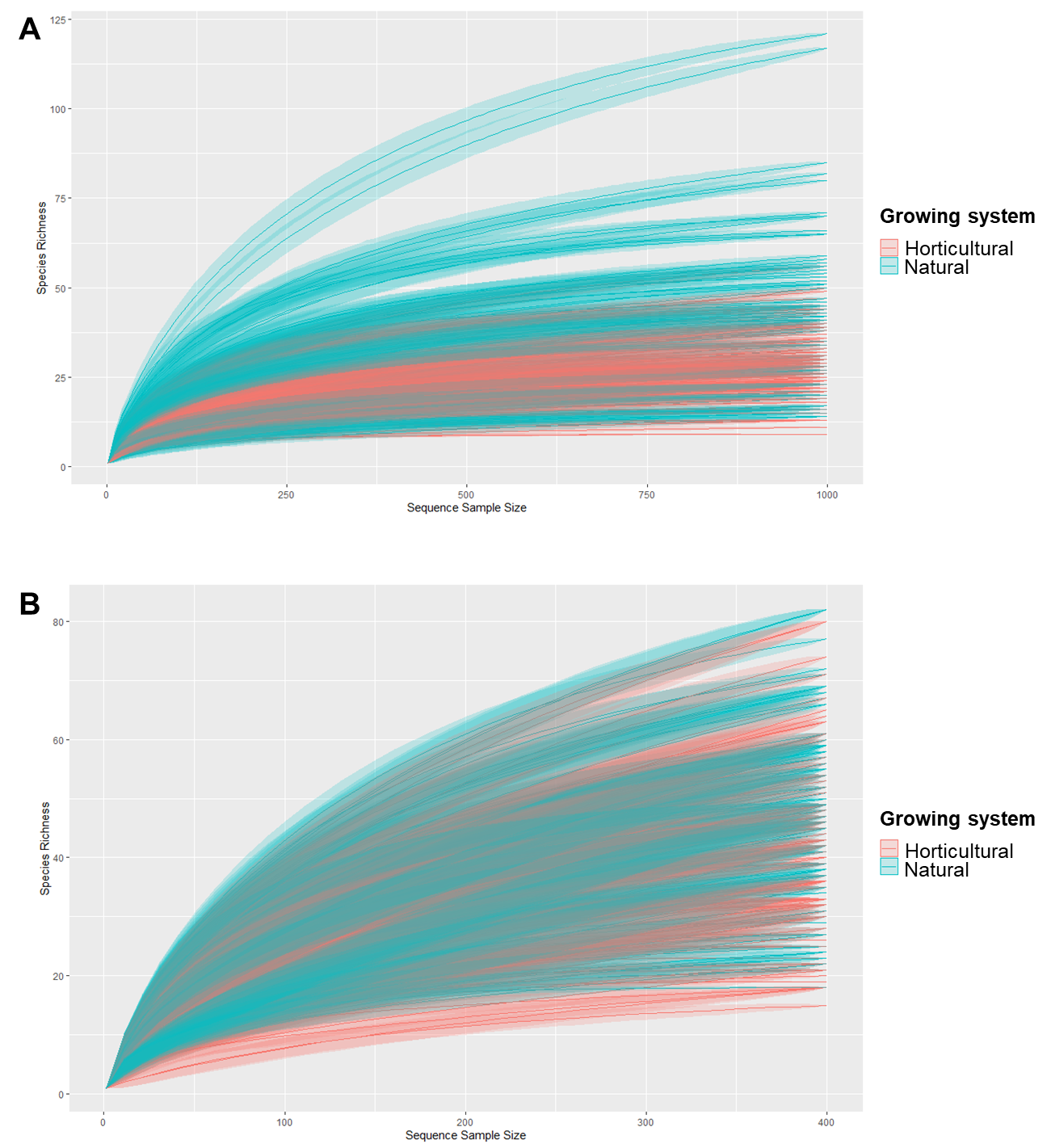
**

**Supplementary** **Figure S1.** Rarefaction curves for 16S rRNA and ITS gene amplicon libraries of naturally grown and horticultural fruits.

**
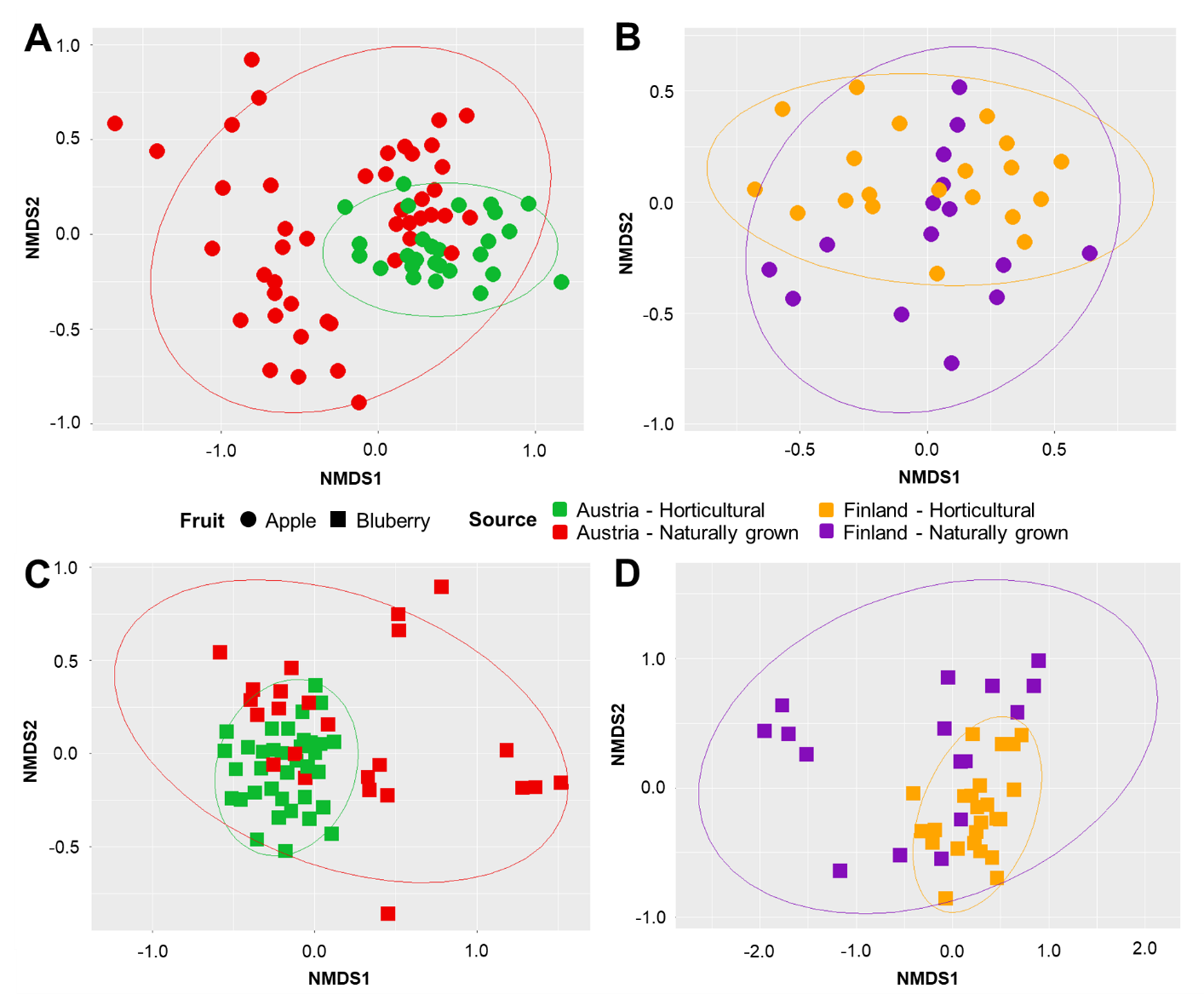
**

**Supplementary** **Figure S2. Community clustering of bacterial composition between of naturally grown and horticultural fruits.** The nonmetric multidimensional scaling (MDS) plots show bacterial communities from apples and blueberries that were obtained in Austria (A and C, respectively) and Finland (B and D, respectively).

**
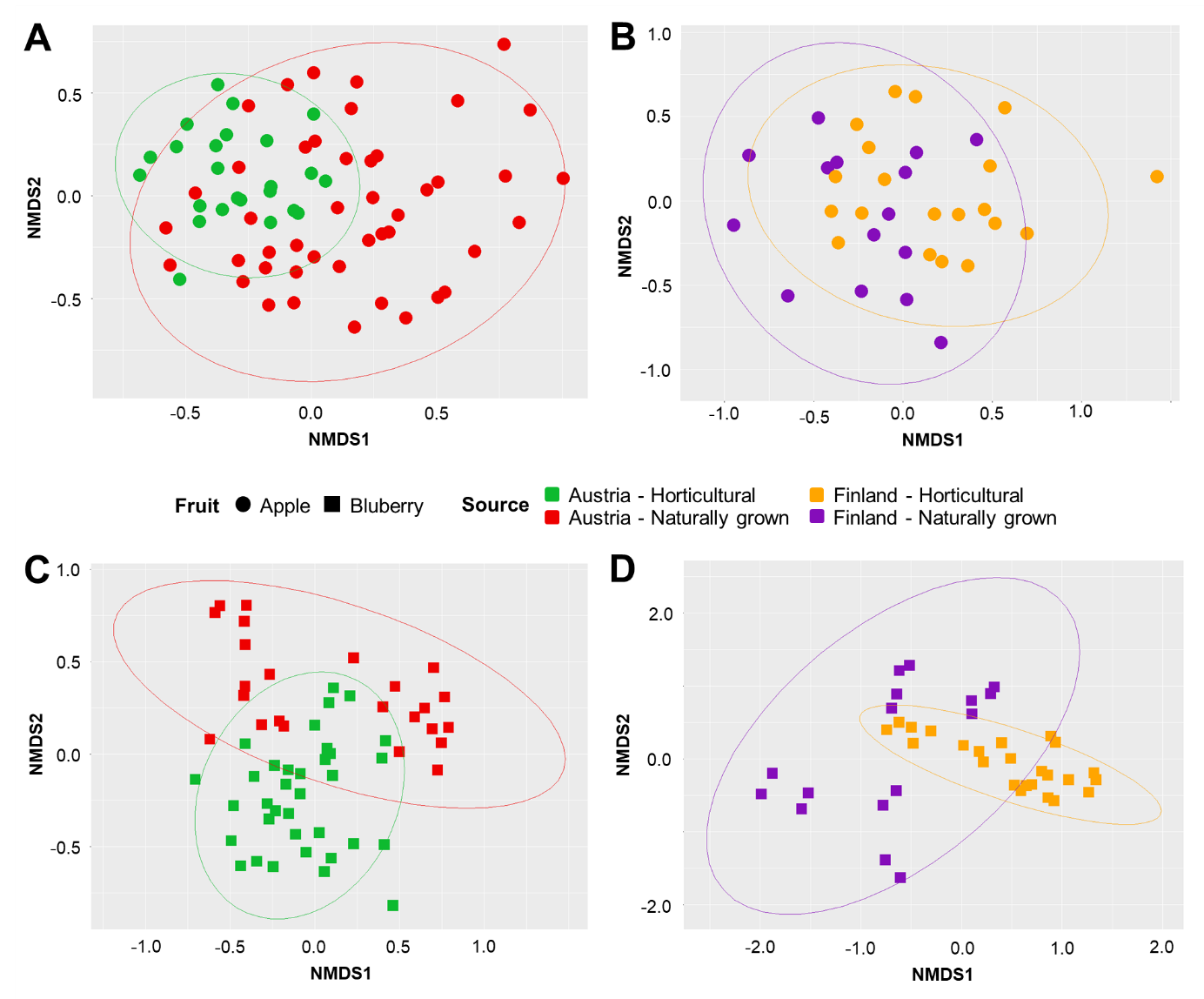
**

**Supplementary** **Figure S3. Community clustering of fungal community compositions of naturally grown and horticultural fruits**. The nonmetric multidimensional scaling (MDS) plots show fungal communities from apples and blueberries that were obtained in Austria (A and C, respectively) and Finland (B and D, respectively).
